# A precise measurement of the $B^0$ meson oscillation frequency

**DOI:** 10.1140/epjc/s10052-016-4250-2

**Published:** 2016-07-21

**Authors:** R. Aaij, C. Abellán Beteta, B. Adeva, M. Adinolfi, A. Affolder, Z. Ajaltouni, S. Akar, J. Albrecht, F. Alessio, M. Alexander, S. Ali, G. Alkhazov, P. Alvarez Cartelle, A. A. Alves Jr, S. Amato, S. Amerio, Y. Amhis, L. An, L. Anderlini, J. Anderson, G. Andreassi, M. Andreotti, J. E. Andrews, R. B. Appleby, O. Aquines Gutierrez, F. Archilli, P. d’Argent, A. Artamonov, M. Artuso, E. Aslanides, G. Auriemma, M. Baalouch, S. Bachmann, J. J. Back, A. Badalov, C. Baesso, W. Baldini, R. J. Barlow, C. Barschel, S. Barsuk, W. Barter, V. Batozskaya, V. Battista, A. Bay, L. Beaucourt, J. Beddow, F. Bedeschi, I. Bediaga, L. J. Bel, V. Bellee, N. Belloli, I. Belyaev, E. Ben-Haim, G. Bencivenni, S. Benson, J. Benton, A. Berezhnoy, R. Bernet, A. Bertolin, M.-O. Bettler, M. van Beuzekom, A. Bien, S. Bifani, P. Billoir, T. Bird, A. Birnkraut, A. Bizzeti, T. Blake, F. Blanc, J. Blouw, S. Blusk, V. Bocci, A. Bondar, N. Bondar, W. Bonivento, S. Borghi, M. Borsato, T. J. V. Bowcock, E. Bowen, C. Bozzi, S. Braun, M. Britsch, T. Britton, J. Brodzicka, N. H. Brook, E. Buchanan, A. Bursche, J. Buytaert, S. Cadeddu, R. Calabrese, M. Calvi, M. Calvo Gomez, P. Campana, D. Campora Perez, L. Capriotti, A. Carbone, G. Carboni, R. Cardinale, A. Cardini, P. Carniti, L. Carson, K. Carvalho Akiba, G. Casse, L. Cassina, L. Castillo Garcia, M. Cattaneo, Ch. Cauet, G. Cavallero, R. Cenci, M. Charles, Ph. Charpentier, M. Chefdeville, S. Chen, S.-F. Cheung, N. Chiapolini, M. Chrzaszcz, X. Cid Vidal, G. Ciezarek, P. E. L. Clarke, M. Clemencic, H. V. Cliff, J. Closier, V. Coco, J. Cogan, E. Cogneras, V. Cogoni, L. Cojocariu, G. Collazuol, P. Collins, A. Comerma-Montells, A. Contu, A. Cook, M. Coombes, S. Coquereau, G. Corti, M. Corvo, B. Couturier, G. A. Cowan, D. C. Craik, A. Crocombe, M. Cruz Torres, S. Cunliffe, R. Currie, C. D’Ambrosio, E. Dall’Occo, J. Dalseno, P. N. Y. David, A. Davis, O. De Aguiar Francisco, K. De Bruyn, S. De Capua, M. De Cian, J. M. De Miranda, L. De Paula, P. De Simone, C.-T. Dean, D. Decamp, M. Deckenhoff, L. Del Buono, N. Déléage, M. Demmer, D. Derkach, O. Deschamps, F. Dettori, B. Dey, A. Di Canto, F. Di Ruscio, H. Dijkstra, S. Donleavy, F. Dordei, M. Dorigo, A. Dosil Suárez, D. Dossett, A. Dovbnya, K. Dreimanis, L. Dufour, G. Dujany, F. Dupertuis, P. Durante, R. Dzhelyadin, A. Dziurda, A. Dzyuba, S. Easo, U. Egede, V. Egorychev, S. Eidelman, S. Eisenhardt, U. Eitschberger, R. Ekelhof, L. Eklund, I. El Rifai, Ch. Elsasser, S. Ely, S. Esen, H. M. Evans, T. Evans, A. Falabella, C. Färber, N. Farley, S. Farry, R. Fay, D. Ferguson, V. Fernandez Albor, F. Ferrari, F. Ferreira Rodrigues, M. Ferro-Luzzi, S. Filippov, M. Fiore, M. Fiorini, M. Firlej, C. Fitzpatrick, T. Fiutowski, K. Fohl, P. Fol, M. Fontana, F. Fontanelli, D. C. Forshaw, R. Forty, M. Frank, C. Frei, M. Frosini, J. Fu, E. Furfaro, A. Gallas Torreira, D. Galli, S. Gallorini, S. Gambetta, M. Gandelman, P. Gandini, Y. Gao, J. García Pardiñas, J. Garra Tico, L. Garrido, D. Gascon, C. Gaspar, R. Gauld, L. Gavardi, G. Gazzoni, D. Gerick, E. Gersabeck, M. Gersabeck, T. Gershon, Ph. Ghez, S. Gianì, V. Gibson, O. G. Girard, L. Giubega, V. V. Gligorov, C. Göbel, D. Golubkov, A. Golutvin, A. Gomes, C. Gotti, M. Grabalosa Gándara, R. Graciani Diaz, L. A. Granado Cardoso, E. Graugés, E. Graverini, G. Graziani, A. Grecu, E. Greening, S. Gregson, P. Griffith, L. Grillo, O. Grünberg, B. Gui, E. Gushchin, Yu. Guz, T. Gys, T. Hadavizadeh, C. Hadjivasiliou, G. Haefeli, C. Haen, S. C. Haines, S. Hall, B. Hamilton, X. Han, S. Hansmann-Menzemer, N. Harnew, S. T. Harnew, J. Harrison, J. He, T. Head, V. Heijne, A. Heister, K. Hennessy, P. Henrard, L. Henry, J. A. Hernando Morata, E. van Herwijnen, M. Heß, A. Hicheur, D. Hill, M. Hoballah, C. Hombach, W. Hulsbergen, T. Humair, N. Hussain, D. Hutchcroft, D. Hynds, M. Idzik, P. Ilten, R. Jacobsson, A. Jaeger, J. Jalocha, E. Jans, A. Jawahery, F. Jing, M. John, D. Johnson, C. R. Jones, C. Joram, B. Jost, N. Jurik, S. Kandybei, W. Kanso, M. Karacson, T. M. Karbach, S. Karodia, M. Kecke, M. Kelsey, I. R. Kenyon, M. Kenzie, T. Ketel, B. Khanji, C. Khurewathanakul, T. Kirn, S. Klaver, K. Klimaszewski, O. Kochebina, M. Kolpin, I. Komarov, R. F. Koopman, P. Koppenburg, M. Kozeiha, L. Kravchuk, K. Kreplin, M. Kreps, G. Krocker, P. Krokovny, F. Kruse, W. Krzemien, W. Kucewicz, M. Kucharczyk, V. Kudryavtsev, A. K. Kuonen, K. Kurek, T. Kvaratskheliya, D. Lacarrere, G. Lafferty, A. Lai, D. Lambert, G. Lanfranchi, C. Langenbruch, B. Langhans, T. Latham, C. Lazzeroni, R. Le Gac, J. van Leerdam, J.-P. Lees, R. Lefèvre, A. Leflat, J. Lefrançois, E. Lemos Cid, O. Leroy, T. Lesiak, B. Leverington, Y. Li, T. Likhomanenko, M. Liles, R. Lindner, C. Linn, F. Lionetto, B. Liu, X. Liu, D. Loh, I. Longstaff, J. H. Lopes, D. Lucchesi, M. Lucio Martinez, H. Luo, A. Lupato, E. Luppi, O. Lupton, N. Lusardi, A. Lusiani, F. Machefert, F. Maciuc, O. Maev, K. Maguire, S. Malde, A. Malinin, G. Manca, G. Mancinelli, P. Manning, A. Mapelli, J. Maratas, J. F. Marchand, U. Marconi, C. Marin Benito, P. Marino, J. Marks, G. Martellotti, M. Martin, M. Martinelli, D. Martinez Santos, F. Martinez Vidal, D. Martins Tostes, A. Massafferri, R. Matev, A. Mathad, Z. Mathe, C. Matteuzzi, A. Mauri, B. Maurin, A. Mazurov, M. McCann, J. McCarthy, A. McNab, R. McNulty, B. Meadows, F. Meier, M. Meissner, D. Melnychuk, M. Merk, E Michielin, D. A. Milanes, M.-N. Minard, D. S. Mitzel, J. Molina Rodriguez, I. A. Monroy, S. Monteil, M. Morandin, P. Morawski, A. Mordà, M. J. Morello, J. Moron, A. B. Morris, R. Mountain, F. Muheim, D. Müller, J. Müller, K. Müller, V. Müller, M. Mussini, B. Muster, P. Naik, T. Nakada, R. Nandakumar, A. Nandi, I. Nasteva, M. Needham, N. Neri, S. Neubert, N. Neufeld, M. Neuner, A. D. Nguyen, T. D. Nguyen, C. Nguyen-Mau, V. Niess, R. Niet, N. Nikitin, T. Nikodem, A. Novoselov, D. P. O’Hanlon, A. Oblakowska-Mucha, V. Obraztsov, S. Ogilvy, O. Okhrimenko, R. Oldeman, C. J. G. Onderwater, B. Osorio Rodrigues, J. M. Otalora Goicochea, A. Otto, P. Owen, A. Oyanguren, A. Palano, F. Palombo, M. Palutan, J. Panman, A. Papanestis, M. Pappagallo, L. L. Pappalardo, C. Pappenheimer, C. Parkes, G. Passaleva, G. D. Patel, M. Patel, C. Patrignani, A. Pearce, A. Pellegrino, G. Penso, M. Pepe Altarelli, S. Perazzini, P. Perret, L. Pescatore, K. Petridis, A. Petrolini, M. Petruzzo, E. Picatoste Olloqui, B. Pietrzyk, T. Pilař, D. Pinci, A. Pistone, A. Piucci, S. Playfer, M. Plo Casasus, T. Poikela, F. Polci, A. Poluektov, I. Polyakov, E. Polycarpo, A. Popov, D. Popov, B. Popovici, C. Potterat, E. Price, J. D. Price, J. Prisciandaro, A. Pritchard, C. Prouve, V. Pugatch, A. Puig Navarro, G. Punzi, W. Qian, R. Quagliani, B. Rachwal, J. H. Rademacker, M. Rama, M. S. Rangel, I. Raniuk, N. Rauschmayr, G. Raven, F. Redi, S. Reichert, M. M. Reid, A. C. dos Reis, S. Ricciardi, S. Richards, M. Rihl, K. Rinnert, V. Rives Molina, P. Robbe, A. B. Rodrigues, E. Rodrigues, J. A. Rodriguez Lopez, P. Rodriguez Perez, S. Roiser, V. Romanovsky, A. Romero Vidal, J. W. Ronayne, M. Rotondo, J. Rouvinet, T. Ruf, P. Ruiz Valls, J. J. Saborido Silva, N. Sagidova, P. Sail, B. Saitta, V. Salustino Guimaraes, C. Sanchez Mayordomo, B. Sanmartin Sedes, R. Santacesaria, C. Santamarina Rios, M. Santimaria, E. Santovetti, A. Sarti, C. Satriano, A. Satta, D. M. Saunders, D. Savrina, S. Schael, M. Schiller, H. Schindler, M. Schlupp, M. Schmelling, T. Schmelzer, B. Schmidt, O. Schneider, A. Schopper, M. Schubiger, M.-H. Schune, R. Schwemmer, B. Sciascia, A. Sciubba, A. Semennikov, A. Sergi, N. Serra, J. Serrano, L. Sestini, P. Seyfert, M. Shapkin, I. Shapoval, Y. Shcheglov, T. Shears, L. Shekhtman, V. Shevchenko, A. Shires, B. G. Siddi, R. Silva Coutinho, L. Silva de Oliveira, G. Simi, M. Sirendi, N. Skidmore, T. Skwarnicki, E. Smith, E. Smith, I. T. Smith, J. Smith, M. Smith, H. Snoek, M. D. Sokoloff, F. J. P. Soler, F. Soomro, D. Souza, B. Souza De Paula, B. Spaan, P. Spradlin, S. Sridharan, F. Stagni, M. Stahl, S. Stahl, S. Stefkova, O. Steinkamp, O. Stenyakin, S. Stevenson, S. Stoica, S. Stone, B. Storaci, S. Stracka, M. Straticiuc, U. Straumann, L. Sun, W. Sutcliffe, K. Swientek, S. Swientek, V. Syropoulos, M. Szczekowski, P. Szczypka, T. Szumlak, S. T’Jampens, A. Tayduganov, T. Tekampe, M. Teklishyn, G. Tellarini, F. Teubert, C. Thomas, E. Thomas, J. van Tilburg, V. Tisserand, M. Tobin, J. Todd, S. Tolk, L. Tomassetti, D. Tonelli, S. Topp-Joergensen, N. Torr, E. Tournefier, S. Tourneur, K. Trabelsi, M. T. Tran, M. Tresch, A. Trisovic, A. Tsaregorodtsev, P. Tsopelas, N. Tuning, A. Ukleja, A. Ustyuzhanin, U. Uwer, C. Vacca, V. Vagnoni, G. Valenti, A. Vallier, R. Vazquez Gomez, P. Vazquez Regueiro, C. Vázquez Sierra, S. Vecchi, M. van Veghel, J. J. Velthuis, M. Veltri, G. Veneziano, M. Vesterinen, B. Viaud, D. Vieira, M. Vieites Diaz, X. Vilasis-Cardona, A. Vollhardt, D. Volyanskyy, D. Voong, A. Vorobyev, V. Vorobyev, C. Voß, J. A. de Vries, R. Waldi, C. Wallace, R. Wallace, J. Walsh, S. Wandernoth, J. Wang, D. R. Ward, N. K. Watson, D. Websdale, A. Weiden, M. Whitehead, G. Wilkinson, M. Wilkinson, M. Williams, M. P. Williams, M. Williams, T. Williams, F. F. Wilson, J. Wimberley, J. Wishahi, W. Wislicki, M. Witek, G. Wormser, S. A. Wotton, S. Wright, K. Wyllie, Y. Xie, Z. Xu, Z. Yang, J. Yu, X. Yuan, O. Yushchenko, M. Zangoli, M. Zavertyaev, L. Zhang, Y. Zhang, A. Zhelezov, A. Zhokhov, L. Zhong, V. Zhukov, S. Zucchelli

**Affiliations:** 1 Centro Brasileiro de Pesquisas Físicas (CBPF), Rio de Janeiro, Brazil; 2 Universidade Federal do Rio de Janeiro (UFRJ), Rio de Janeiro, Brazil; 3 Center for High Energy Physics, Tsinghua University, Beijing, China; 4 LAPP, Université Savoie Mont-Blanc, CNRS/IN2P3, Annecy-Le-Vieux, France; 5 Clermont Université, Université Blaise Pascal, CNRS/IN2P3, LPC, Clermont-Ferrand, France; 6 CPPM, Aix-Marseille Université, CNRS/IN2P3, Marseille, France; 7 LAL, Université Paris-Sud, CNRS/IN2P3, Orsay, France; 8 LPNHE, Université Pierre et Marie Curie, Université Paris Diderot, CNRS/IN2P3, Paris, France; 9 I. Physikalisches Institut, RWTH Aachen University, Aachen, Germany; 10 Fakultät Physik, Technische Universität Dortmund, Dortmund, Germany; 11 Max-Planck-Institut für Kernphysik (MPIK), Heidelberg, Germany; 12 Physikalisches Institut, Ruprecht-Karls-Universität Heidelberg, Heidelberg, Germany; 13 School of Physics, University College Dublin, Dublin, Ireland; 14 Sezione INFN di Bari, Bari, Italy; 15 Sezione INFN di Bologna, Bologna, Italy; 16 Sezione INFN di Cagliari, Cagliari, Italy; 17 Sezione INFN di Ferrara, Ferrara, Italy; 18 Sezione INFN di Firenze, Firenze, Italy; 19 Laboratori Nazionali dell’INFN di Frascati, Frascati, Italy; 20 Sezione INFN di Genova, Genova, Italy; 21 Sezione INFN di Milano Bicocca, Milan, Italy; 22 Sezione INFN di Milano, Milan, Italy; 23 Sezione INFN di Padova, Padua, Italy; 24 Sezione INFN di Pisa, Pisa, Italy; 25 Sezione INFN di Roma Tor Vergata, Rome, Italy; 26 Sezione INFN di Roma La Sapienza, Rome, Italy; 27 Henryk Niewodniczanski Institute of Nuclear Physics Polish Academy of Sciences, Kraków, Poland; 28 Faculty of Physics and Applied Computer Science, AGH-University of Science and Technology, Kraków, Poland; 29 National Center for Nuclear Research (NCBJ), Warsaw, Poland; 30 Horia Hulubei National Institute of Physics and Nuclear Engineering, Bucharest-Magurele, Romania; 31 Petersburg Nuclear Physics Institute (PNPI), Gatchina, Russia; 32 Institute of Theoretical and Experimental Physics (ITEP), Moscow, Russia; 33 Institute of Nuclear Physics, Moscow State University (SINP MSU), Moscow, Russia; 34 Institute for Nuclear Research of the Russian Academy of Sciences (INR RAN), Moscow, Russia; 35 Budker Institute of Nuclear Physics (SB RAS), Novosibirsk State University, Novosibirsk, Russia; 36 Institute for High Energy Physics (IHEP), Protvino, Russia; 37 Universitat de Barcelona, Barcelona, Spain; 38 Universidad de Santiago de Compostela, Santiago de Compostela, Spain; 39 European Organization for Nuclear Research (CERN), Geneva, Switzerland; 40 Ecole Polytechnique Fédérale de Lausanne (EPFL), Lausanne, Switzerland; 41 Physik-Institut, Universität Zürich, Zurich, Switzerland; 42 Nikhef National Institute for Subatomic Physics, Amsterdam, The Netherlands; 43 Nikhef National Institute for Subatomic Physics, VU University Amsterdam, Amsterdam, The Netherlands; 44 NSC Kharkiv Institute of Physics and Technology (NSC KIPT), Kharkiv, Ukraine; 45 Institute for Nuclear Research of the National Academy of Sciences (KINR), Kyiv, Ukraine; 46 University of Birmingham, Birmingham, UK; 47 H.H. Wills Physics Laboratory, University of Bristol, Bristol, UK; 48 Cavendish Laboratory, University of Cambridge, Cambridge, UK; 49 Department of Physics, University of Warwick, Coventry, UK; 50 STFC Rutherford Appleton Laboratory, Didcot, UK; 51 School of Physics and Astronomy, University of Edinburgh, Edinburgh, UK; 52 School of Physics and Astronomy, University of Glasgow, Glasgow, UK; 53 Oliver Lodge Laboratory, University of Liverpool, Liverpool, UK; 54 Imperial College London, London, UK; 55 School of Physics and Astronomy, University of Manchester, Manchester, UK; 56 Department of Physics, University of Oxford, Oxford, UK; 57 Massachusetts Institute of Technology, Cambridge, MA USA; 58 University of Cincinnati, Cincinnati, OH USA; 59 University of Maryland, College Park, MD USA; 60 Syracuse University, Syracuse, NY USA; 61 Pontifícia Universidade Católica do Rio de Janeiro (PUC-Rio), Rio de Janeiro, Brazil; 62 Institute of Particle Physics, Central China Normal University, Wuhan, Hubei China; 63 Departamento de Fisica, Universidad Nacional de Colombia, Bogota, Colombia; 64 Institut für Physik, Universität Rostock, Rostock, Germany; 65 National Research Centre Kurchatov Institute, Moscow, Russia; 66 Yandex School of Data Analysis, Moscow, Russia; 67 Instituto de Fisica Corpuscular (IFIC), Universitat de Valencia-CSIC, Valencia, Spain; 68 Van Swinderen Institute, University of Groningen, Groningen, The Netherlands

## Abstract

The oscillation frequency, $\Delta m_d$, of $B^0$ mesons is measured using semileptonic decays with a $D^-$ or $D^{*-}$ meson in the final state. The data sample corresponds to 3.0$fb^{-1}$ of *pp* collisions, collected by the LHCb experiment at centre-of-mass energies $\sqrt{s}$ = 7 and 8$\mathrm \,TeV$. A combination of the two decay modes gives $\Delta m_d = (505.0 \pm 2.1 \pm 1.0) \mathrm \,ns^{-1}$, where the first uncertainty is statistical and the second is systematic. This is the most precise single measurement of this parameter. It is consistent with the current world average and has similar precision.

## Introduction

Flavour oscillation, or mixing, of neutral meson systems gives mass eigenstates that are different from flavour eigenstates. In the $${B} ^0$$–$${\overline{B}{}} {}^0$$ system, the mass difference between mass eigenstates, $$\Delta m_{{d}}$$, is directly related to the square of the product of the CKM matrix elements $${V_{{t} {b}}} $$ and $${V_{{t} {d}}^*} $$, and is therefore sensitive to fundamental parameters of the Standard Model, as well as to non-perturbative strong-interaction effects and the square of the top quark mass [1]. Measurements of mixing of neutral $$B $$ mesons were published for the first time by UA1 [2] and ARGUS [3]. Measurements of $${B} ^0$$–$${\overline{B}{}} {}^0$$ mixing have been performed by CLEO [4], experiments at LEP and SLC [5], experiments at the Tevatron [6, 7], the $$B $$ Factories experiments [8, 9] and, most recently, at LHCb [10–12]. The combined world average value for the mass difference, $${\Delta m_{{d}}} =(510 \pm 3)$$ $$\mathrm{\,ns^{-1}}$$, has a relative precision of 0.6 % [13]. This paper reports a measurement of $$\Delta m_{{d}}$$ based on $${{B} ^0} \!\rightarrow {{D} ^-} {\mu ^+} {{\nu } _\mu } X$$ and $${{B} ^0} \!\rightarrow {{D} ^{*-}} {\mu ^+} {{\nu } _\mu } X$$ decays,^1^ where *X* indicates any additional particles that are not reconstructed. The data sample used for this measurement was collected at LHCb during LHC Run 1 at $$\sqrt{s}$$ = 7 (8)TeV in 2011 (2012), corresponding to integrated luminosities of 1.0 (2.0)$$bad hbox^{-1}$$.

The relatively high branching fraction for semileptonic decays of ${B} ^0$ mesons, along with the highly efficient lepton identification and flavour tagging capabilities at LHCb, results in abundant samples of ${{B} ^0} \!\rightarrow {D} ^{(*)-} {\mu ^+} {{\nu } _\mu } X$ decays, where the flavour of the ${B} ^0$ meson at the time of production and decay can be inferred. In addition, the decay time *t* of ${B} ^0$ mesons can be determined with adequate resolution, even though the decay is not fully reconstructed, because of the potential presence of undetected particles. It is therefore possible to precisely measure $\Delta m_{{d}}$ as the frequency of matter-antimatter oscillations in a time-dependent analysis of the decay rates of unmixed and mixed events,

1 $$\begin{aligned} N^\mathrm{unmix}(t)&\equiv N({{B} ^0} \!\rightarrow {D} ^{(*)-} {\mu ^+} {{\nu } _\mu } X)(t) \propto e^{-{\Gamma _{{d}}} t} \left[ 1 + \cos ({\Delta m_{{d}}} t)\right] \ ,\nonumber \\ N^\mathrm{mix}(t)&\equiv N({{B} ^0} \rightarrow {{\overline{B}{}} {}^0} \!\rightarrow {D} ^{(*)+} {\mu ^-} {{\overline{\nu }} _\mu } X)(t) \propto e^{-{\Gamma _{{d}}} t} \left[ 1 - \cos ({\Delta m_{{d}}} t) \right] \ , \end{aligned}$$

where the state assignment is based on the flavours of the ${B} ^0$ meson at production and decay, which may be the same (unmixed) or opposite (mixed). In Eq. 1, ${\Gamma _{{d}}} =1/\tau _{{{B} ^0}}$ is the decay width of the ${B} ^0$ meson, $\tau _{{{B} ^0}}$ being its lifetime. Also, in Eq. 1 the difference in the decay widths of the mass eigenstates, $\Delta {\Gamma _{{d}}},$ and $C\!P$ violation in mixing are neglected, due to their negligible impact on the results. The flavour asymmetry between unmixed and mixed events is

2 $$\begin{aligned} A(t) = \frac{N^\mathrm{unmix}(t) - N^\mathrm{mix} (t)}{N^\mathrm{unmix}(t) + N^\mathrm{mix}(t)} = \cos ({\Delta m_{{d}}} t) \ . \end{aligned}$$

A description of the LHCb detector and the datasets used in this measurement is given in Sect. 2. Section 3 presents the selection criteria, the flavour tagging algorithms, and the method chosen to reconstruct the ${B} ^0$ decay time. The fitting strategy and results are described in Sect. 4. A summary of the systematic uncertainties is given in Sect. 5, and conclusions are reported in Sect. 6.

## Detector and simulation

The LHCb detector [14, 15] is a single-arm forward spectrometer covering the pseudorapidity range $$2<\eta <5$$, designed for the study of particles containing $$b $$ or $$c $$ quarks. The detector includes a high-precision tracking system consisting of a silicon-strip vertex detector surrounding the *pp* interaction region, a large-area silicon-strip detector located upstream of a dipole magnet with a bending power of about $$4\mathrm{\,Tm}$$, and three stations of silicon-strip detectors and straw drift tubes placed downstream of the magnet. The tracking system provides a measurement of momentum, $$p$$, of charged particles with a relative uncertainty that varies from 0.5 % at low momentum to 1.0 % at 200$${{\mathrm {\, GeV}/c}}$$. The minimum distance of a track to a primary vertex (PV), the impact parameter (IP), is measured with a resolution of $$(15+29/p_\mathrm{T}){\,\upmu \mathrm{m}} $$, where $$p_\mathrm{T}$$ is the component of the momentum transverse to the beam, in $${{\mathrm {\, GeV}/c}}$$. Different types of charged hadrons are distinguished using information from two ring-imaging Cherenkov (RICH) detectors. Photons, electrons and hadrons are identified by a calorimeter system consisting of scintillating-pad and preshower detectors, an electromagnetic calorimeter and a hadronic calorimeter. Muons are identified by a system composed of alternating layers of iron and multiwire proportional chambers.

The online event selection is performed by a trigger [16], which consists of a hardware stage, based on information from the calorimeter and muon systems, followed by a software stage, which applies a full event reconstruction. Candidate events are first required to pass the hardware trigger, which selects muons with a transverse momentum $$p_\mathrm{T} >1.48{{\mathrm {\, GeV}/c}}$$ in the 7TeVdata or $$p_\mathrm{T} >1.76{{\mathrm {\, GeV}/c}}$$ in the 8TeVdata. The software trigger requires a two-, three- or four-track secondary vertex, where one of the tracks is identified as a muon, with a significant displacement from the primary *pp* interaction vertices. At least one charged particle must have a transverse momentum $$p_\mathrm{T} > 1.7{{\mathrm {\, GeV}/c}}$$ and be inconsistent with originating from a PV. As it will be explained later, the software trigger selection introduces a bias on the $$\Delta m_{{d}}$$ measurement, which is corrected for. A multivariate algorithm [17] is used for the identification of secondary vertices consistent with the decay of a $$b $$ hadron.

The method chosen to reconstruct the ${B} ^0$ decay time relies on Monte Carlo simulation. Simulation is also used to estimate the main background sources and to verify the fit model. In the simulation, *pp* collisions are generated using Pythia  [18, 19] with a specific LHCb configuration [20]. Decays of hadronic particles are described by EvtGen  [21], in which final-state radiation is generated using Photos  [22]. The interaction of the generated particles with the detector, and its response, are implemented using the Geant4 toolkit [23, 24] as described in Ref. [25]. Large samples of mixtures of semileptonic decays resulting in a ${D} ^-$ or a ${D} ^{*-}$ meson in the final state were simulated and the assumptions used to build these samples are assessed in the evaluation of systematic uncertainties.

## Event selection

For charged particles used to reconstruct signal candidates, requirements are imposed on track quality, momentum, transverse momentum, and impact parameter with respect to any PV. Tracks are required to be identified as muons, kaons or pions. The charm mesons are reconstructed through the ${{D} ^-} \!\rightarrow {{K} ^+} {{\pi } ^-} {{\pi } ^-}$ decay, or through the ${{D} ^{*-}} \!\rightarrow {{\overline{D}} {}^0} {{\pi } ^-}$, ${{\overline{D}} {}^0} \!\rightarrow {{K} ^+} {{\pi } ^-}$ decay chain. The masses of the reconstructed ${D} ^-$ and ${\overline{D}} {}^0$ mesons should be within $70{{\mathrm {\,MeV/}c^2}}$ and $40{{\mathrm {\,MeV/}c^2}}$ of their known values [13], while the mass difference between the reconstructed ${D} ^{*-}$ and ${\overline{D}} {}^0$ mesons should lie between $140{{\mathrm {\,MeV/}c^2}}$ and $155{{\mathrm {\,MeV/}c^2}}$. For ${D} ^-$ and ${\overline{D}} {}^0$ candidates, the scalar sum of the $p_\mathrm{T}$ of the daughter tracks should be above $1800{{\mathrm {\,MeV/}c}}$. A good quality vertex fit is required for the ${D} ^-$, ${\overline{D}} {}^0$, and ${D} ^{*-}$ candidates, and for the $D^{(*)-}\mu ^+$ combinations. When more than one combination is found in an event, the one with the smallest vertex ${\chi ^2}$ (hereafter referred to as the $B$ candidate) is chosen. The reconstructed vertices of ${D} ^-$, ${\overline{D}} {}^0$, and $B$ candidates are required to be significantly displaced from their associated PV, where the associated PV is that which has the smallest ${\chi ^2}$ increase when adding the candidate. For ${D} ^-$ and ${\overline{D}} {}^0$ candidates, a large IP with respect to the associated PV is required in order to suppress charm mesons promptly produced in *pp* collisions. The momentum of the $B$ candidate, and its flight direction measured using the PV and the $B$ vertex positions, are required to be aligned. These selection criteria reduce to the per-mille level or lower the contribution of ${D} ^{(*)-}$ decays where the charmed meson originates from the PV. The invariant mass of the $B$ candidate is required to be in the range $[3.0,5.2]{{\mathrm {\,GeV/}c^2}}$.

Backgrounds from $B \rightarrow {{{{J}/{\psi }}}}X$ decays, where one of the muons from the ${{{{J}/{\psi }}}}\rightarrow {\mu ^+} {\mu ^-}$ decay is correctly identified and the other misidentified as a pion and used to reconstruct a ${D} ^{(*)-}$, are suppressed by applying a veto around the ${{{{J}/{\psi }}}}$ mass. Similarly, a veto around the ${\varLambda } ^+_{c}$ mass is applied to suppress semileptonic decays of the ${\varLambda } ^0_{b}$ baryon, in which the proton of the subsequent ${\varLambda } ^+_{c}$ decay into $p K^- \pi ^+$ is misidentified as a pion.

The dominant background is due to $${{B} ^+} \!\rightarrow {D} ^{(*)-} {\mu ^+} {{\nu } _\mu } X$$ decays, where additional particles coming from the decay of higher charm resonances, or from multi-body decays of $${B} ^+$$ mesons, are neglected. The fractions of $${B} ^+$$ decays in the $${D} ^-$$ and $${D} ^{*-}$$ samples are expected to be 13 and 10 %, based on the branching fractions of signal and background, with uncertainties at the 10 % level. This background is reduced by using a multivariate discriminant based on a boosted decision tree (BDT) algorithm [26, 27], which exploits information on the $$B$$ candidate, kinematics of the higher charm resonances and isolation criteria for tracks and composite candidates in the $$B$$ decay chain. Training of the BDT classifier is carried out using simulation samples of $${{B} ^0} \!\rightarrow {{D} ^{*-}} {\mu ^+} {{\nu } _\mu } X$$ signal and $${{B} ^+} \!\rightarrow {{D} ^{*-}} {\mu ^+} {{\nu } _\mu } X$$ background. The variables used as input for the BDT classifier are described in the Appendix. Only candidates with BDT output larger than $$-0.12$$ ($$-0.16$$) are selected in the 2011 (2012) data sample for the $${{B} ^0} \!\rightarrow {{D} ^-} {\mu ^+} {{\nu } _\mu } X$$ mode. The BDT output is required to be larger than $$-0.3$$ in both 2011 and 2012 data samples for the $${{B} ^0} \!\rightarrow {{D} ^{*-}} {\mu ^+} {{\nu } _\mu } X$$ mode. The impact of this requirement on signal efficiency and background retention can be seen in Fig. 3. The background from $${B} ^+$$ decays is reduced by 70 % in both modes. Combinatorial background is evaluated by using reconstructed candidates in the $${D} ^{(*)-}$$ signal mass sidebands. Backgrounds due to decays of $${B} ^0_{s} $$ and $${\varLambda } ^0_{b} $$ into similar final states to those of the signal are studied through simulations.

The decay time of the ${B} ^0$ meson is calculated as $t = {(M_{{{B} ^0}} \cdot L)}/{(p_\mathrm{rec}\cdot c / k)}$, where $M_{{{B} ^0}}$ is the mass of the ${B} ^0$, taken from Ref. [13], *L* is the measured decay length and $p_\mathrm{rec}$ is the magnitude of the visible momentum, measured from the ${D} ^{(*)-}$ meson and the muon. The correction factor *k* is determined from simulation by dividing the visible ${B} ^0$ momentum by its true value and taking the average, $k = \langle {p_\mathrm{rec}}/{p_\mathrm{true}} \rangle$. This correction represents the dominant source of uncertainty in the determination of the decay time of the ${B} ^0$ meson for $t > 1.5 \mathrm{\,ps}$. Since the *k*-factor depends strongly on the decay kinematics, it is parametrised by a fourth-order polynomial as a function of the visible mass of the ${B} ^0$ candidate as explained in the Appendix.

The $${B} ^0$$ flavour at production is determined by using information from the other $$b $$ hadron present in the event. The decision of flavour tagging algorithms [28] based on the charge of leptons, kaons and of an inclusively reconstructed detached vertex, is used for the $${{B} ^0} \!\rightarrow {{D} ^{*-}} {\mu ^+} {{\nu } _\mu } X$$ channel. In the $${{B} ^0} \!\rightarrow {{D} ^-} {\mu ^+} {{\nu } _\mu } X$$ channel, which is subject to a larger $${{B} ^+} $$ background contamination, the decision of the tagging algorithm based on the detached vertex is excluded in order to avoid spurious background asymmetries. The statistical uncertainty on $$\Delta m_{{d}}$$ decreases as $$\mathcal{{T}}^{-1/2}$$ where the tagging power is defined as $$\mathcal{{T}}={{{{\varepsilon _\mathrm{tag}}}(1-2\omega )^2}}$$, where $${{\varepsilon _\mathrm{tag}}}$$ is the tagging efficiency and $${\omega }$$ is the mistag rate. To increase the statistical precision, the events are grouped into four tagging categories of increasing predicted mistag probability $$\eta $$, defined by $$\eta \in [0,0.25]$$, [0.25, 0.33], [0.33, 0.41], [0.41, 0.47]. The mistag probability $$\eta $$ is evaluated for each $$B$$ candidate from event and taggers properties and was calibrated on data using control samples [28]. The average mistag rates for signal and background are taken as free parameters when fitting for $$\Delta m_{{d}}$$. The combined tagging power [28] for the $${{B} ^0} \!\rightarrow {{D} ^-} {\mu ^+} {{\nu } _\mu } X$$ mode is $$(2.38\pm 0.05)$$ % and $$(2.46\pm 0.04)$$ % in 2011 and 2012. For the $${{B} ^0} \!\rightarrow {{D} ^{*-}} {\mu ^+} {{\nu } _\mu } X$$ mode, the tagging power in 2011 and 2012 is $$(2.55\pm 0.07)$$ % and $$(2.32\pm 0.04)$$ %.

## Fit strategy and results

The fit proceeds as follows. First, ${D} ^{(*)-}$ mesons originating from semileptonic ${B} ^0$ or ${B} ^+$ decays are separated from the background coming from combinations of tracks not associated to a charm meson decay, by a fit to the invariant mass distributions of the selected candidates. This fit assigns to each event a covariance-weighted quantity *sWeight*, which is used in the subsequent fits to subtract statistically the contribution of the background by means of the ${sPlot}$ procedure [29]. Then, the contribution of ${D} ^{(*)-}$ from ${B} ^+$ decays is determined in a fit to the distributions of the BDT classifier output weighted by signal *sWeights*. Next, a cut is applied on the BDT output in order to suppress the ${B} ^+$ background, the mass distributions are fitted again, and new *sWeights* are determined. Finally, the oscillation frequency $\Delta m_{{d}}$ is determined by a fit to the decay time distribution of unmixed and mixed candidates, weighted for the signal *sWeights* determined in the previous step.

An extended binned maximum likelihood fit to the data distributions is performed for each stage, simultaneously for the four tagging categories defined above. Data samples collected in 2011 and 2012 are treated separately.

**Fig. 1:**
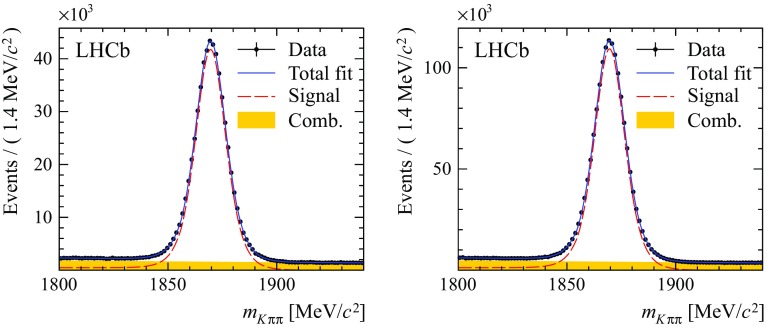
Distribution of $m_{K\pi \pi }$ for the ${{B} ^0} \!\rightarrow {{D} ^-} {\mu ^+} {{\nu } _\mu } X$ candidates in (*left*) 2011 and (*right*) 2012 data. Projections of the fit function are superimposed (*blue continuous line*) for the full PDF and its components: (*red dashed line*) signal ${D} ^-$ from ${B} ^0$ or ${B} ^+$ decays and (*filled yellow area*) combinatorial background

Figure 1 shows the results of the fits to the ${D} ^-$ candidate mass distributions for ${{B} ^0} \!\rightarrow {{D} ^-} {\mu ^+} {{\nu } _\mu } X$ candidates. In these fits, the distributions of ${D} ^-$ from ${B} ^0$ and ${B} ^+$ decays are summed as they are described by the same probability density function (PDF): the sum of two Gaussian functions and a Crystal Ball function [30]. The yields corresponding to the ${D} ^-$ peak are $(5.30 \pm 0.02)\times 10^5$ and $(1.393 \pm 0.003)\times 10^6$ in 2011 and 2012 data, respectively. The combinatorial background, which contributes typically 6 % under the ${D} ^-$ peak, is modelled with an exponential distribution.

**Fig. 2:**
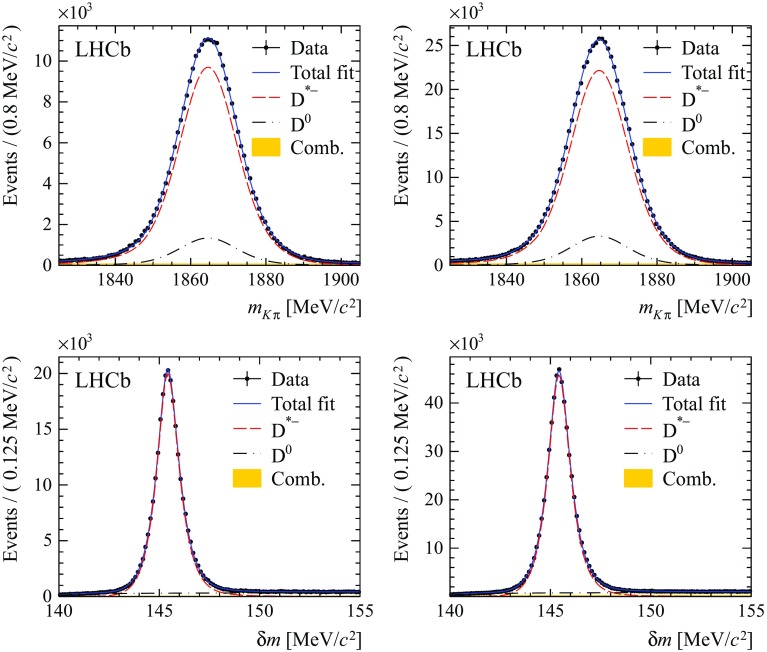
Distributions of (*top*) $$m_{K\pi }$$ and (*bottom*) $$\delta m$$ for $${{B} ^0} \!\rightarrow {{D} ^{*-}} {\mu ^+} {{\nu } _\mu } X$$ candidates in (*left*) 2011 and (*right*) 2012 data. Projections of the fit function are superimposed for (*blue continuous line*) the full PDF and its components: (*red dashed line*) signal $${D} ^{*-}$$ from $${B} ^0$$ or $${B} ^+$$ decays, (*black dashed-dotted line*) $${\overline{D}} {}^0$$ from $$B $$ and (*filled yellow area*) combinatorial backgrounds

For the $${{B} ^0} \!\rightarrow {{D} ^{*-}} {\mu ^+} {{\nu } _\mu } X$$ samples, a simultaneous fit to the distributions of the $$K^+\pi ^-$$ invariant mass, $$m_{K^+\pi ^-}$$, and the invariant mass difference of $${K^+\pi ^-\pi ^-}$$ and $${K^+\pi ^-}$$ combinations, $$\delta m = m_{K^+\pi ^-\pi ^-} - m_{K^+\pi ^-}$$, is performed. Three different components are considered: the signal $${D} ^*$$ from $${B} ^0$$ or $${B} ^+$$ decays and two background sources. The PDF for the mass distributions of $${D} ^*$$ from $$B $$ decays is defined by the sum of two Gaussian functions and a Crystal Ball function in the $$m_{K^+\pi ^-}$$ mass projection and by two Gaussian functions and a Johnson function [31] in the $$\delta m$$ mass projection. Background candidates containing a $${\overline{D}} {}^0$$ originating from a $$b $$ hadron decay without an intermediate $$D^*$$ resonance, which contribute about 15 % in the full $$\delta m$$ mass range, are described by the same distribution as that of the signal for $$m_{K^+\pi ^-}$$, and by an empirical function based on a phase-space distribution for $$\delta m$$. A combinatorial background component which contributes typically 0.8 % under the $${D} ^*$$ peak is modelled with an exponential distribution for $$m_{K^+\pi ^-}$$ and the same empirical distribution for $$\delta m$$ as used for the $${\overline{D}} {}^0$$ background. All parameters that describe signal and background shapes are allowed to vary freely in the invariant mass fits. The results of the 2011 and 2012 fits for these parameters are compatible within the statistical uncertainties. Figure 2 shows the results of the fit to the $${{B} ^0} \!\rightarrow {{D} ^{*-}} {\mu ^+} {{\nu } _\mu } X$$ samples, projected onto the two mass observables. The yields corresponding to the $${D} ^*$$ peak are $$(2.514 \pm 0.006) \times 10^5$$ and $$(5.776 \pm 0.009)\times 10^5$$ in 2011 and 2012 data.

**Fig. 3:**
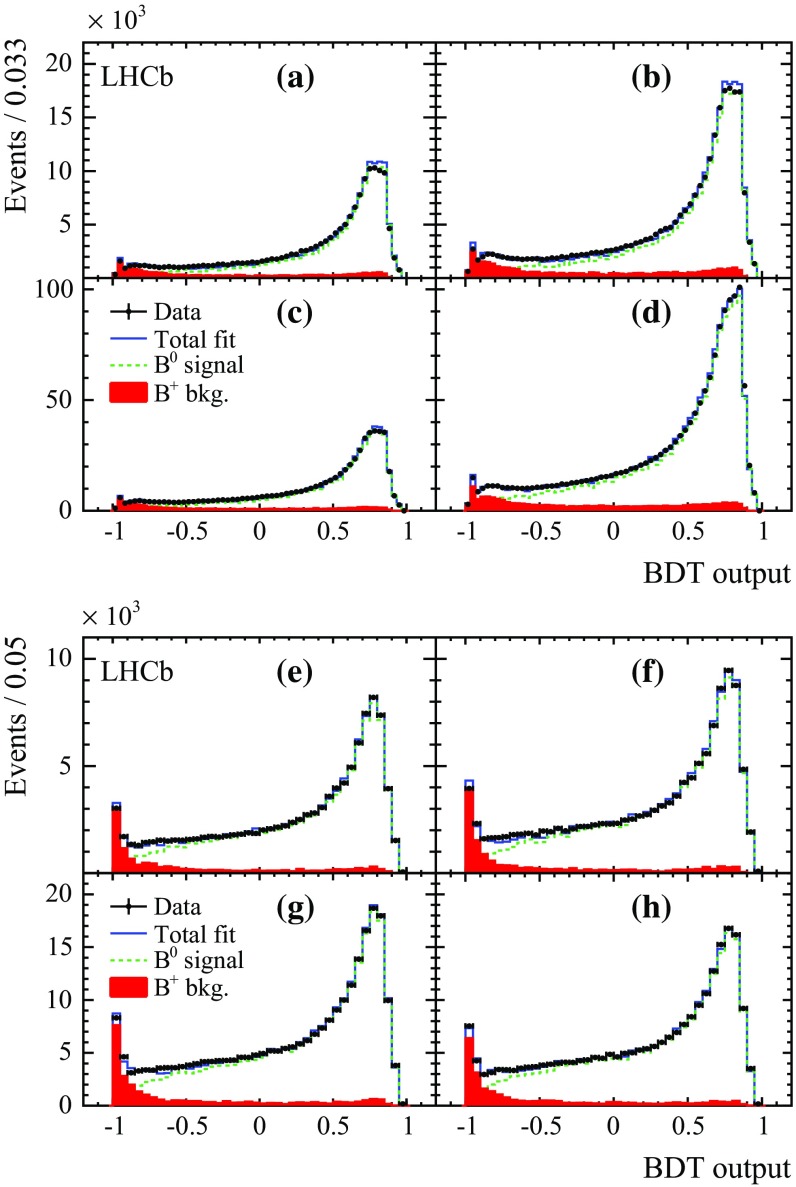
Fits to the output of the ${B} ^+$ veto BDT for (*top four plots*) ${{B} ^0} \!\rightarrow {{D} ^-} {\mu ^+} {{\nu } _\mu } X$ and (*bottom four plots*) ${{B} ^0} \!\rightarrow {{D} ^{*-}} {\mu ^+} {{\nu } _\mu } X$ in 2012 data, for each tagging category. The *filled red histogram*, the *dashed green line*, and the *continuous blue line* correspond to background, signal, and total templates, respectively. The average mistag fraction per category increases when going from **a** to **d** and **e** to **h**.

The fraction of ${B} ^+$ background in data, $\alpha _{{{B} ^+}}$, is determined with good precision by fitting the distribution of the BDT classifier, where templates for signal and ${B} ^+$ background are obtained from simulation. Fits are performed separately in tagging categories for 2011 and 2012 data, giving fractions of ${B} ^+$ of 6 and 3 % on average for the ${{B} ^0} \!\rightarrow {{D} ^-} {\mu ^+} {{\nu } _\mu } X$ and the ${{B} ^0} \!\rightarrow {{D} ^{*-}} {\mu ^+} {{\nu } _\mu } X$ modes with relative variation of the order of 10 % between samples. The results of the fits to 2012 data for both modes are given in Fig. 3. Limited knowledge of the exclusive decays used to build the simulation templates leads to systematic uncertainties of 0.5 and 0.4 % on the ${B} ^+$ fractions for ${{B} ^0} \!\rightarrow {{D} ^-} {\mu ^+} {{\nu } _\mu } X$ and ${{B} ^0} \!\rightarrow {{D} ^{*-}} {\mu ^+} {{\nu } _\mu } X$. In the decay time fit, the ${B} ^+$ fractions are kept fixed. The statistical and systematic uncertainties on $\alpha _{{{B} ^+}}$ lead to a systematic uncertainty on $\Delta m_{{d}}$, which is reported in Sect. 5.

The oscillation frequency $\Delta m_{{d}}$ is determined from a binned maximum likelihood fit to the distribution of the ${B} ^0$ decay time *t* of candidates classified as mixed ($q=-1$) or unmixed ($q=1$) according to the flavour of the ${B} ^0$ meson at production and decay time.

The total PDF for the fit is given by

3 $$\begin{aligned} \mathcal{P}(t,q) = { \mathcal S}(t,q) + \alpha _{_{{B} ^+}} \mathcal{B}^{+}(t,q) \, , \end{aligned}$$

where the time distributions for signal and background are given by

4 $$\begin{aligned} \mathcal{S}(t,q)= & {} \mathcal{N} e^{-{\Gamma _{{d}}} t}\Big ( 1 + q (1 - 2{\omega }_\mathrm{sig}) \cos {\Delta m_{{d}}} t \Big ) \, ,\\ \mathcal{B^+}(t,q)= & {} \mathcal{N}_{_{{B} ^+}} e^{-\Gamma _u t}\left( \frac{1+q}{2}-q {\omega }_{{{B} ^+}}\right) \, . \nonumber \end{aligned}$$

Here $\mathcal{N}$ and $\mathcal{N_{_{{B} ^+}}}$ are normalisation factors, and $\Gamma _d$ and $\Gamma _u$ are fixed in the fit to their world average values [13], where $\Gamma _u = 1/{\tau _{{{B} ^+}}}$, with $\tau _{{{B} ^+}}$ being the lifetime of the ${B} ^+$ meson. The mistag fractions for signal and ${B} ^+$ components, ${\omega }_\mathrm{sig}$ and ${\omega }_{{{B} ^+}}$, vary freely in the fit. To account for the time resolution, both distributions in Eq. 4 are convolved with a resolution model that takes into account uncertainties on both the decay length and the momentum. The distributions used in the fit are therefore obtained by a double convolution. The contribution accounting for the decay length resolution is described by a triple Gaussian function with an effective width corresponding to a time resolution of $75\mathrm{\,fs}$, as determined from simulation. The contribution accounting for the uncertainty on the momentum is described by the distribution of $p_\mathrm{rec}/(k \cdot p_\mathrm{true})$, obtained from the simulation. This second convolution is dominant above 1.5 $\mathrm{\,ps}$. Finally, the function $\mathcal {P}$ is multiplied by an acceptance function *a*(*t*) to account for the effect of the trigger and offline selection and reconstruction. The acceptance is described by a sum of cubic spline polynomials [32], which may be different for signal and ${B} ^+$ background. The ratios between spline coefficients of the ${B} ^+$ background acceptance and those of the signal acceptance are fixed to the values predicted by simulation. The spline coefficients for signal are then determined for each tagging category directly from the tagged time-dependent fit to data.

The fitting strategy is validated with simulation. A bias is observed in the $\Delta m_{{d}}$ value, due to a correlation between the decay time and its resolution, which is not taken into account when parameterizing the signal shape. Simulation shows that this correlation is introduced by the requirements of the software trigger and offline selection on the impact parameters of ${D} ^-$ and ${\overline{D}} {}^0$ with respect to the PV. Values for this bias, of up to 4$\mathrm{\,ns^{-1}}$ with a 10 % uncertainty, are determined for each mode and for each year by fitting the true and corrected time distributions and taking the differences between the resulting values of $\Delta m_{{d}}$. The uncertainty on the bias is treated as a systematic uncertainty on $\Delta m_{{d}}$.

**Fig. 4:**
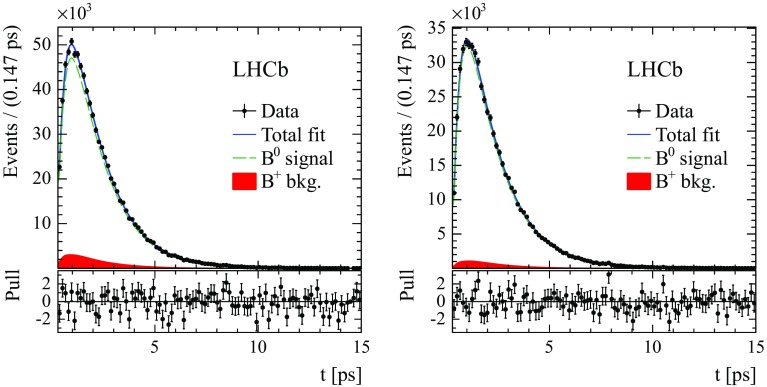
Decay time distributions for (*left*) ${{B} ^0} \!\rightarrow {{D} ^-} {\mu ^+} {{\nu } _\mu } X$ and (*right*) ${{B} ^0} \!\rightarrow {{D} ^{*-}} {\mu ^+} {{\nu } _\mu } X$ in the category with lowest mistag in 2012 data

**Fig. 5:**
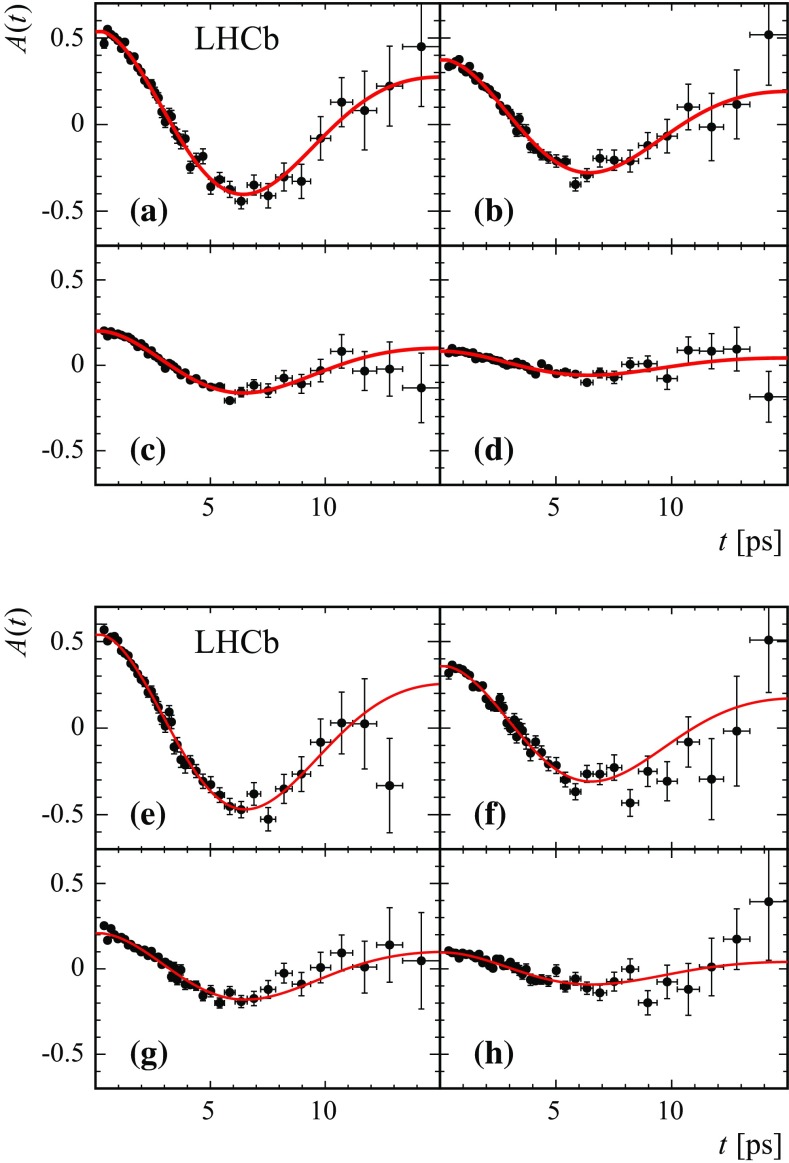
Mixing asymmetry projections in the four tagging categories for (*top plots*) ${{B} ^0} \!\rightarrow {{D} ^-} {\mu ^+} {{\nu } _\mu } X$ and (*bottom plots*) ${{B} ^0} \!\rightarrow {{D} ^{*-}} {\mu ^+} {{\nu } _\mu } X$ for 2011 data. The average mistag per category increases when going from **a** to **d**, and from **e** to **h**

**Fig. 6:**
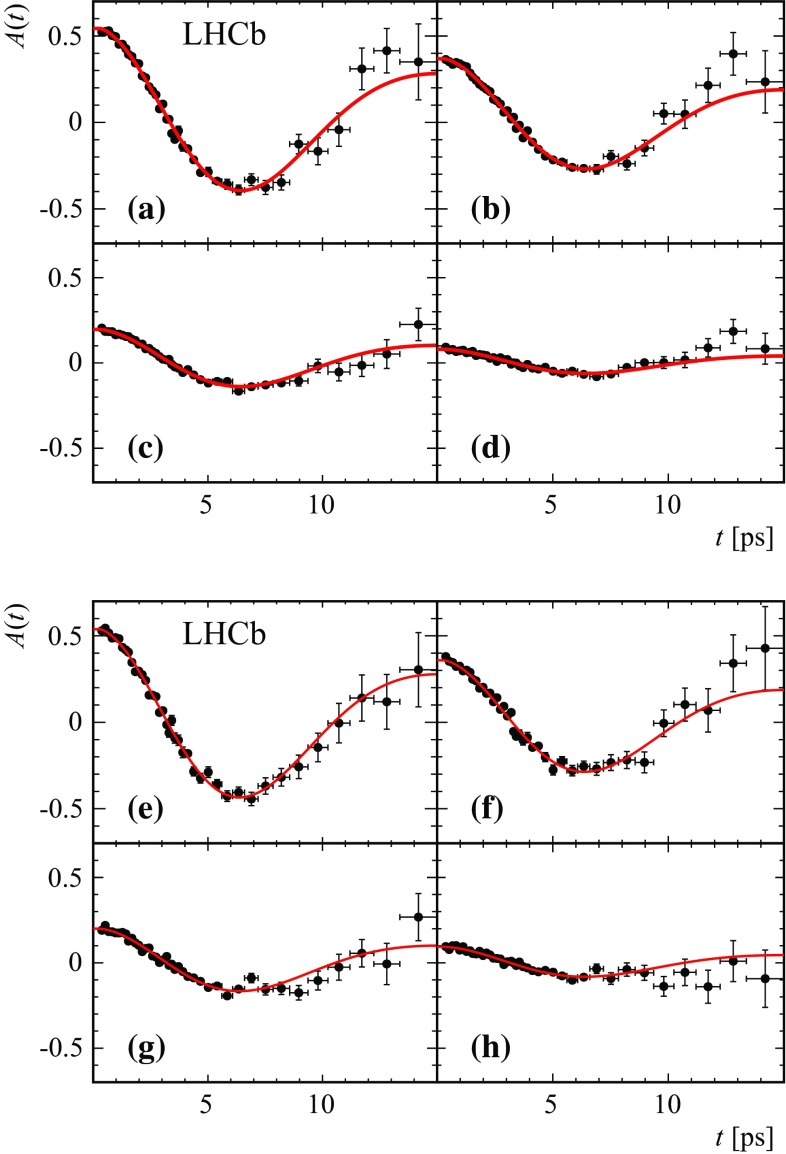
Mixing asymmetry projections in the four tagging categories for (*top plots*) ${{B} ^0} \!\rightarrow {{D} ^-} {\mu ^+} {{\nu } _\mu } X$ and (*bottom plots*) ${{B} ^0} \!\rightarrow {{D} ^{*-}} {\mu ^+} {{\nu } _\mu } X$ for 2012 data. The average mistag per category increases when going from **a** to **d**, and from **e** to **h**

The values of $\Delta m_{{d}}$, obtained from the time-dependent fit and corrected for the fit bias, are reported in Table 1. Systematic uncertainties are discussed below. The four independent $\Delta m_{{d}}$ values are compatible within statistical uncertainties. Figure 4 shows the fit projections for the decay time distributions for the candidates in the category with lowest mistag rate in 2012 data. The time-dependent asymmetries for the ${{B} ^0} \!\rightarrow {{D} ^-} {\mu ^+} {{\nu } _\mu } X$ and ${{B} ^0} \!\rightarrow {{D} ^{*-}} {\mu ^+} {{\nu } _\mu } X$ modes in 2011 and 2012 data are shown in Figs. 5 and 6. Fits are also performed in subsamples of different track multiplicity, number of primary vertices, magnet polarity, run periods, and muon charges. Statistically compatible results are obtained in all cases. A combination of the two $\Delta m_{{d}}$ determinations, including systematic uncertainties, is given in Sect. 6.

**Table 1:** Results for $\Delta m_{{d}}$ measured in each mode for 2011 and 2012 data separately, for the total sample, and for the combination of the two modes. The quoted uncertainties for the separate samples are statistical only. For the total samples and the combination, they refer to statistical and total systematic uncertainties, respectively

Mode	2011 sample	2012 sample	Total sample
	$\Delta m_{{d}}$ ($\mathrm{\,ns^{-1}}$)	$\Delta m_{{d}}$ ($\mathrm{\,ns^{-1}}$)	$\Delta m_{{d}}$ ($\mathrm{\,ns^{-1}}$)
${{B} ^0} \!\rightarrow {{D} ^-} {\mu ^+} {{\nu } _\mu } X$	$506.2\pm 5.1$	$505.2\pm 3.1$	$505.5\pm 2.7\pm 1.1$
${{B} ^0} \!\rightarrow {{D} ^{*-}} {\mu ^+} {{\nu } _\mu } X$	$497.5\pm 6.1$	$508.3\pm 4.0$	$504.4\pm 3.4\pm 1.0$
Combination			$505.0\pm 2.1\pm 1.0$

## Systematic uncertainties

The contribution of each source of systematic uncertainty is evaluated by using a large number of parameterized simulations. The difference between the default $\Delta m_{{d}}$ value and the result obtained when repeating the fits after having adjusted the inputs to those corresponding to the systematic variation under test, is taken as a systematic uncertainty. Systematic uncertainties are summarized in Table 2.

**Table 2:** Sources of systematic uncertainties on $\Delta m_{{d}}$, separated into those that are correlated and uncorrelated between the two decay channels ${{B} ^0} \!\rightarrow {{D} ^-} {\mu ^+} {{\nu } _\mu } X$ and ${{B} ^0} \!\rightarrow {{D} ^{*-}} {\mu ^+} {{\nu } _\mu } X$

Source of uncertainty	${{B} ^0} \!\rightarrow {{D} ^-} {\mu ^+} {{\nu } _\mu } X$ ($\mathrm{\,ns^{-1}}$)		${{B} ^0} \!\rightarrow {{D} ^{*-}} {\mu ^+} {{\nu } _\mu } X$ ($\mathrm{\,ns^{-1}}$)	
	Uncorrelated	Correlated	Uncorrelated	Correlated
${B} ^+$ background	0.4	0.1	0.4	–
Other backgrounds	–	0.5	–	–
*k*-factor distribution	0.4	0.5	0.3	0.6
Other fit-related	0.5	0.4	0.3	0.5
Total	0.8	0.8	0.6	0.8

### Background from $\mathbf {B^+}$

The fraction of ${B} ^+$ background is estimated from data with a very small statistical uncertainty. A variation, within their uncertainties, of the branching fractions of semileptonic ${B} ^0$ decays resulting in a ${D} ^{*-}$ or ${D} ^-$ in the final state gives systematic uncertainties on the ${B} ^+$ fractions of 0.5 and 0.4 % for ${{B} ^0} \!\rightarrow {{D} ^-} {\mu ^+} {{\nu } _\mu } X$ and ${{B} ^0} \!\rightarrow {{D} ^{*-}} {\mu ^+} {{\nu } _\mu } X$. The resulting uncertainty on $\Delta m_{{d}}$ is 0.1$\mathrm{\,ns^{-1}}$ in ${{B} ^0} \!\rightarrow {{D} ^-} {\mu ^+} {{\nu } _\mu } X$ and is negligible for ${{B} ^0} \!\rightarrow {{D} ^{*-}} {\mu ^+} {{\nu } _\mu } X$. In the default fit, the decay time acceptance ratio of the ${B} ^0$ and the ${B} ^+$ components is taken from simulation. The time acceptance is to a large extent due to the cut on the ${D} ^0$ impact parameter. A possible systematic effect due to an incorrect determination of the acceptance ratio from simulation is estimated by fitting events, generated with the default signal and background acceptances, with an acceptance ratio determined by using a tighter ${D} ^0$ IP cut than the default. This gives an uncertainty of 0.4$\mathrm{\,ns^{-1}}$ on both decay modes. The above systematic uncertainties are considered as uncorrelated between the two channels.

The uncertainty on $$\Delta m_{{d}}$$ from the resolution on the $${{B} ^+} $$ decay length is 0.1$$\mathrm{\,ns^{-1}}$$ in the $${{B} ^0} \!\rightarrow {{D} ^-} {\mu ^+} {{\nu } _\mu } X$$ channel and is negligible in the $${{B} ^0} \!\rightarrow {{D} ^{*-}} {\mu ^+} {{\nu } _\mu } X$$ channel.

### Other backgrounds

The impact of the knowledge of backgrounds due to semileptonic $${B} ^0_{s} $$ decays with $${D} ^{(*)-}$$ in the final state is estimated by varying their contributions within the uncertainties on their branching fractions. This effect has a negligible impact on $$\Delta m_{{d}}$$ for both channels. For the $${{B} ^0} \!\rightarrow {{D} ^-} {\mu ^+} {{\nu } _\mu } X$$ channel, there is an additional contribution from $${{B} ^0_{s}} \rightarrow {{D} ^-_{s}} {\mu ^+} {{\nu } _\mu } $$ decays, where a kaon in the $${{D} ^-_{s}} \rightarrow {{K} ^-} {{K} ^+} {{\pi } ^-} $$ decay is misidentified as a pion, which gives an 8 % contribution due to $${D} ^-_{s} $$ peaking under the $${D} ^-$$ mass. A difference in $$\Delta m_{{d}}$$ of 0.5$$\mathrm{\,ns^{-1}}$$ is observed.

The $${{\varLambda } ^0_{b}} \rightarrow {{{n}}}{{D} ^{*-}} {\mu ^+} \nu _{\mu }$$ decay has not been observed. However, because of the similar final state, it can be mistaken for $${{B} ^+} $$ background, since neither of them exhibits oscillatory behaviour. Dedicated simulated samples are generated by assuming colour suppression with respect to signal, and are used to estimate a signal contamination of 0.2 % from $${\varLambda } ^0_{b} $$ decays, with $$100\,\%$$ uncertainty, which gives a negligible effect on $$\Delta m_{{d}}$$.

Small contributions from $${B} \rightarrow D^{(*)-} D_s^{+}X$$ decays, with the $${{D} ^+_{s}} $$ decaying semileptonically give an uncertainty of 0.2$$\mathrm{\,ns^{-1}}$$ on $$\Delta m_{{d}}$$ in the $${{B} ^0} \!\rightarrow {{D} ^-} {\mu ^+} {{\nu } _\mu } X$$ mode, and a negligible effect for the $${{B} ^0} \!\rightarrow {{D} ^{*-}} {\mu ^+} {{\nu } _\mu } X$$ mode.

### The $\varvec{k}$-factor

Two main sources of systematic uncertainty are related to the *k*-factor. The first, due to possible differences in the $B$ momentum spectrum between simulation and data, is studied by comparing the $B$ momentum in ${{B} ^+} \!\rightarrow {{{{J}/{\psi }}}}{{K} ^+}$ decays in data and simulation, and reweighting signal simulation to estimate the effect on the *k*-factor distribution and therefore on $\Delta m_{{d}}$. The systematic uncertainties on $\Delta m_{{d}}$ from this effect for ${{B} ^0} \!\rightarrow {{D} ^-} {\mu ^+} {{\nu } _\mu } X$ and ${{B} ^0} \!\rightarrow {{D} ^{*-}} {\mu ^+} {{\nu } _\mu } X$ are 0.3$\mathrm{\,ns^{-1}}$ and 0.5$\mathrm{\,ns^{-1}}$. The second source, related to the uncertainties on the measurements of the branching fractions for the exclusive modes which are used to build the simulated samples, is evaluated by varying the branching fractions of exclusive decays one at a time by one standard deviation, and reweighting the corresponding *k*-factor distribution. An uncertainty of 0.4$\mathrm{\,ns^{-1}}$ is obtained for both ${{B} ^0} \!\rightarrow {{D} ^-} {\mu ^+} {{\nu } _\mu } X$ and ${{B} ^0} \!\rightarrow {{D} ^{*-}} {\mu ^+} {{\nu } _\mu } X$ channels. The systematic uncertainties from the *k*-factor correction are taken to be correlated between the two channels.

The systematic uncertainties on $\Delta m_{{d}}$ from the finite number of events in the simulation sample used to compute the *k*-factor corrections are 0.3 and 0.4$\mathrm{\,ns^{-1}}$ (${{B} ^0} \!\rightarrow {{D} ^-} {\mu ^+} {{\nu } _\mu } X$) and 0.2 and 0.3$\mathrm{\,ns^{-1}}$ (${{B} ^0} \!\rightarrow {{D} ^{*-}} {\mu ^+} {{\nu } _\mu } X$) for the 2011 and 2012 samples, respectively.

### Other systematic uncertainties

Possible differences between data and simulation in the resolution on the ${B} ^0$ flight distance are evaluated by using the results of a study reported in Ref. [33], and scaling the widths of the triple Gaussian function by a factor 1.5 with respect to the default. Uncertainties of 0.3$\mathrm{\,ns^{-1}}$ and 0.5$\mathrm{\,ns^{-1}}$ on $\Delta m_{{d}}$ are obtained for ${{B} ^0} \!\rightarrow {{D} ^-} {\mu ^+} {{\nu } _\mu } X$ and ${{B} ^0} \!\rightarrow {{D} ^{*-}} {\mu ^+} {{\nu } _\mu } X$. Both channels are affected by the same discrepancy between data and simulation; thus these systematic uncertainties are taken as correlated.

Since all parameters are allowed to vary freely in the invariant mass fits, the uncertainties from the invariant mass model are small. As a cross-check, when the fits are repeated using the *sWeights* determined without splitting the mass fits in tagging categories, negligible variation in $\Delta m_{{d}}$ is found. Signal and background mistag probabilities are free parameters in the fit, and therefore no systematic uncertainty is associated to them.

Asymmetries in the production of neutral and charged $B$ mesons, in tagging efficiency and mistag probabilities, and in the reconstruction of the final state are neglected in the $\Delta m_{{d}}$ fits. Also, the ${B} ^0$ semileptonic $C\!P$ asymmetry $a_\mathrm{sl}^d$ is assumed to be zero. The systematic uncertainty on $\Delta m_{{d}}$ arising from these assumptions is studied using parameterized simulations with the asymmetries set to zero, to their measured values, and to random variations from their central values within the uncertainties [34]. The resulting uncertainty on $\Delta m_{{d}}$ is found to be negligible.

The bias in $$\Delta m_{{d}}$$ from the correlation between the decay time and its resolution is determined using the simulation. The dependence of $$\Delta m_{{d}}$$ on possible differences between data and simulation has already been considered above by varying the composition of the simulation sample used to construct the *k*-factor distribution. Since the bias is related to the cut on the $$D $$ meson IP with respect to the PV, the fits are repeated with a *k*-factor distribution obtained with a tighter cut on the IP, and the difference with respect to the default is taken as the systematic uncertainty. The systematic uncertainties (0.5 and 0.3$$\mathrm{\,ns^{-1}}$$ for $${{B} ^0} \!\rightarrow {{D} ^-} {\mu ^+} {{\nu } _\mu } X$$ and $${{B} ^0} \!\rightarrow {{D} ^{*-}} {\mu ^+} {{\nu } _\mu } X$$, respectively) related to the bias are considered as uncorrelated between the channels, as they are determined from different simulation samples and the time-biasing cuts, responsible for the systematic uncertainty on the bias, are different for the two channels.

The knowledge of the length scale of the LHCb experiment is limited by the uncertainties from the metrology measurements of the silicon-strip vertex detector. This was evaluated in the context of the $\Delta m_s$ measurement and found to be 0.022 % [33]. This translates into an uncertainty on $\Delta m_{{d}}$ of 0.1$\mathrm{\,ns^{-1}}$. The uncertainty on the knowledge of the momentum scale is determined by reconstructing the masses of various particles and is found to be 0.03 % [35]. This uncertainty results in a 0.2 $\mathrm{\,ns^{-1}}$ uncertainty in $\Delta m_{{d}}$ in both modes. Both uncertainties are considered correlated across the two channels.

Effects due to the choice of the binning scheme and fitting ranges are found to be negligible.

## Summary and conclusion

A combined value of $\Delta m_{{d}}$ is obtained as a weighted average of the four measurements performed in ${{B} ^0} \!\rightarrow {{D} ^-} {\mu ^+} {{\nu } _\mu } X$ and ${{B} ^0} \!\rightarrow {{D} ^{*-}} {\mu ^+} {{\nu } _\mu } X$ in the years 2011 and 2012. First, the 2011 and 2012 results for each decay mode are averaged according to their statistical uncertainties. The combined results are shown in the last column of Table 1. Then, the resulting $\Delta m_{{d}}$ values of each mode are averaged taking account of statistical and uncorrelated systematic uncertainties. The correlated systematic uncertainty is added in quadrature to the resulting uncertainty. The combined result is shown in the last row of Table 1.

In conclusion, the oscillation frequency, $$\Delta m_{{d}}$$, in the $${B} ^0$$–$${\overline{B}{}} {}^0$$ system is measured in semileptonic $${B} ^0$$ decays using data collected in 2011 and 2012 at LHCb. The decays $${{B} ^0} \!\rightarrow {{D} ^-} {\mu ^+} {{\nu } _\mu } X$$ and $${{B} ^0} \!\rightarrow {{D} ^{*-}} {\mu ^+} {{\nu } _\mu } X$$ are used, where the $$D $$ mesons are reconstructed in Cabibbo-favoured decays $${{D} ^-} \!\rightarrow {{K} ^+} {{\pi } ^-} {{\pi } ^-} $$ and $${{D} ^{*-}} \!\rightarrow {{\overline{D}} {}^0} {{\pi } ^-} $$, with $${{\overline{D}} {}^0} \!\rightarrow {{K} ^+} {{\pi } ^-} $$. A combined $$\Delta m_{{d}}$$ measurement is obtained,

$$\begin{aligned} {\Delta m_{{d}}} = \left( 505.0 \pm 2.1 \mathrm {\,\left( stat\right) } \pm 1.0 \mathrm {\,\left( syst\right) } \right) \mathrm{\,ns^{-1}}\ , \end{aligned}$$

which is compatible with previous LHCb results and the world average [13]. This is the most precise single measurement of this quantity, with a total uncertainty similar to the current world average.

## Figures and Tables

**Fig. 7:**
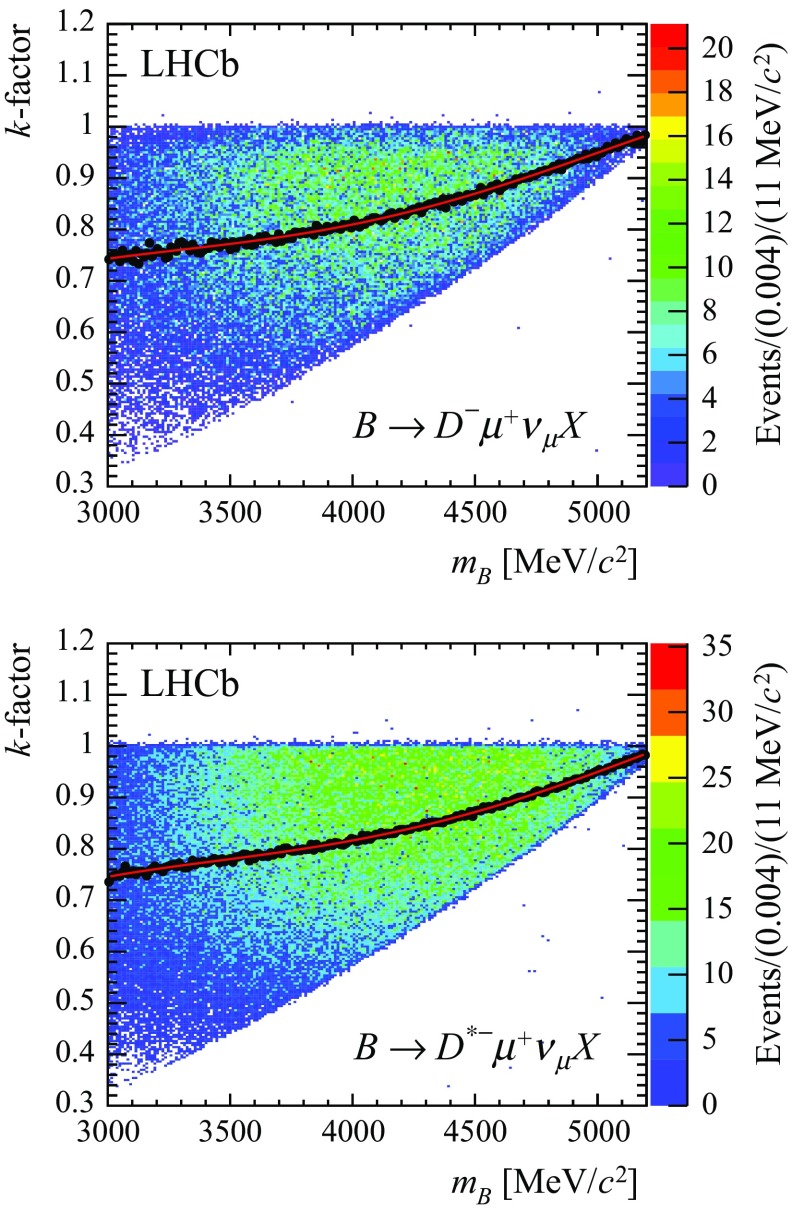
The *k*-factor distribution and the average *k*-factor (*black points*) as a function of the visible mass of the $B$ candidate, in samples of simulated (*top*) ${{B} ^0} \!\rightarrow {{D} ^-} {\mu ^+} {{\nu } _\mu } X$ and (*bottom*) ${{B} ^0} \!\rightarrow {{D} ^{*-}} {\mu ^+} {{\nu } _\mu } X$ decays. Polynomial fits to the average *k*-factor are also shown as a *solid* (*red*) *line*

## Appendix

### BDT classifier

The variables used as input for the BDT classifier are the following:

- Visible mass of the $B$ candidate, $m_{B} \equiv m(D^{(*)-}{\mu ^+})$
- Corrected mass [36], defined as $m_\mathrm{corr}=\sqrt{m_{B}^{2} + p_{T}(B)^{2}} +p_{T}(B)$, where $p_{T}(B)$ is the visible momentum of the $B$ candidate transverse to its flight direction; the $B$ flight direction is measured using the primary vertex and $B$ vertex positions
- Angle between the visible momentum of the $B$ candidate and its flight direction
- Impact parameter, $\mathrm{{IP}}(\pi , D)$, with respect to the decay vertex of the ${D} ^-$ (${\overline{D}} {}^0$), of the track with the smallest impact parameter with respect to the $B$ candidate
- Smallest vertex ${\chi ^2}$ of the combination of the ${D} ^-$ (${D} ^{*-}$) with any other track, and the invariant mass of this combination
- Cone isolation $I = \frac{p_T(B)}{p_T(B) + \sum _{i}p_{T,i}}$, where the sum is computed over tracks which satisfy $\sqrt{\delta \eta _i^{2} +\delta \phi _i^{2}}<1$, $\delta \eta _i$ and $\delta \phi _i$ being the difference in pseudorapidity and in polar angle $\phi$ between the track and the $B$ candidate
- Track isolation variables, used to discriminate tracks originating from the $$B $$ vertex from those originating elsewhere:
  - Number of nearby tracks [37], computed for each track in the $B$ decay chain
  - The output of an isolation BDT [37] estimated for the $B$ candidate
  - A second isolation BDT, similar to the previous, which exploits a different training strategy and additional variables, computed for tracks originating from ${D} ^-$ (${\overline{D}} {}^0$) decays, those coming from the $B$ decay, and all tracks in the decay chain.

The TMVA package [38], used to train and test the classifier, ranks the input variables according to their discriminating power between signal and background.

### Distributions of the *k*-factor

Figure 7 shows distributions of the *k*-factor as a function of the visible mass of the $B$ candidate, as obtained with samples of simulated signal events. In each plot, the average *k*-factor and the result of a polynomial fit are also shown.

## Footnotes

1 The inclusion of charge-conjugate processes is implied throughout.
